# Outcome Prediction of Spontaneous Supratentorial Intracerebral Hemorrhage after Surgical Treatment Based on Non-Contrast Computed Tomography: A Multicenter Study

**DOI:** 10.3390/jcm12041580

**Published:** 2023-02-16

**Authors:** Kangwei Zhang, Xiang Zhou, Qian Xi, Xinyun Wang, Baoqing Yang, Jinxi Meng, Ming Liu, Ningxin Dong, Xiaofen Wu, Tao Song, Lai Wei, Peijun Wang

**Affiliations:** 1 Department of Radiology, Tongji Hospital, Tongji University School of Medicine, Shanghai 200065, China; 2 Department of Radiology, Shanghai East Hospital, Tongji University School of Medicine, Shanghai 200120, China; 3 Department of Radiology, Xinhua Hospital, School of Medicine, Shanghai Jiao Tong University, Shanghai 200092, China; 4 Department of Information, Tongji Hospital, Tongji University School of Medicine, Shanghai 200065, China; 5 SenseTime Research, Shanghai 200233, China

**Keywords:** cerebral hemorrhage, surgical procedures, prognosis, machine learning, radiomics

## Abstract

This study aims to explore the value of a machine learning (ML) model based on radiomics features and clinical features in predicting the outcome of spontaneous supratentorial intracerebral hemorrhage (sICH) 90 days after surgery. A total of 348 patients with sICH underwent craniotomy evacuation of hematoma from three medical centers. One hundred and eight radiomics features were extracted from sICH lesions on baseline CT. Radiomics features were screened using 12 feature selection algorithms. Clinical features included age, gender, admission Glasgow Coma Scale (GCS), intraventricular hemorrhage (IVH), midline shift (MLS), and deep ICH. Nine ML models were constructed based on clinical feature, and clinical features + radiomics features, respectively. Grid search was performed on different combinations of feature selection and ML model for parameter tuning. The averaged receiver operating characteristics (ROC) area under curve (AUC) was calculated and the model with the largest AUC was selected. It was then tested using multicenter data. The combination of lasso regression feature selection and logistic regression model based on clinical features + radiomics features had the best performance (AUC: 0.87). The best model predicted an AUC of 0.85 (95%CI, 0.75–0.94) on the internal test set and 0.81 (95%CI, 0.64–0.99) and 0.83 (95%CI, 0.68–0.97) on the two external test sets, respectively. Twenty-two radiomics features were selected by lasso regression. The second-order feature gray level non-uniformity normalized was the most important radiomics feature. Age is the feature with the greatest contribution to prediction. The combination of clinical features and radiomics features using logistic regression models can improve the outcome prediction of patients with sICH 90 days after surgery.

## 1. Introduction

Spontaneous Intracerebral Hemorrhage (sICH) refers to parenchymal hemorrhage caused by non-traumatic rupture of the cerebral artery. Decompressive craniectomy, minimally invasive surgery, and craniotomy evacuation of hematoma are the main surgical treatments for sICH. Craniotomy evacuation of hematoma can swiftly and effectively remove hematoma, and reduce the mass effect and cytotoxic effect, consequently lowering intracranial pressure and avoiding the development of cerebral hernia, which is a crucial way to preserve patients’ lives [1].

A comparison between early surgery and conservative treatment in two sizable clinical randomized controlled trials of SICH (STICH and STICH II) [2,3] revealed no discernible benefit. Due to severe coma or brain herniation in the two aforementioned tests which decreased mortality in the conservative treatment group, current pertinent studies and guidelines point to a significant crossover rate between the conservative and surgical treatment groups. Additionally, patients in a state of coma and those who were in danger of cerebral herniation were excluded, even though in these situations, surgery might be a lifesaver [1]. In conclusion, there is a solid argument for early surgical treatment even though there is not enough evidence to support it. Surgery may be advantageous for patients with ICH but requires more individualized consideration. Preoperative evaluation is essential for clarifying the surgical efficacy and optimizing treatment. However, there is a lack of accurate prognostic evaluation methods for sICH after surgery.

NCCT can quickly and accurately detect sICH, which is convenient to scan and sensitive to bleeding. It is the first choice for emergency suspected sICH [4]. Our previous study found that NCCT imaging features such as IVH, MLS, and deep ICH were closely related to the postoperative prognosis of patients with sICH [5]. In addition, some studies have pointed out that the special signs found on the NCCT, such as spot sign, vortex sign, hypodensity sign, and black hole sign, are related to hematoma enlargement [6,7,8,9]. Radiomics refers to the high-throughput extraction of quantitative radiomics features from medical images and the use of statistical methods to select them, so as to find out the imaging information with important value for disease diagnosis, efficacy evaluation, and prognosis prediction [10]. ML can complete various tasks such as disease diagnosis, classification, and prognosis prediction through the analysis and processing of big data [11]. Seyedmehdi Payabvash et al. [12] published a research abstract in the journal Stroke. They used ML classifiers to predict the 90-day Modified Rankin Scale (mRS) Score of patients with ICH based on radiomics and compared the performance of different feature selection algorithms and ML classifiers. They found that the combination of radiomics features with clinical features improved model performance. The application of radiomics in the prognosis prediction of ICH faces many problems. The main reasons include the selection of the best algorithm for the model, the lack of external datasets to verify the robustness of the model, and the poor interpretability of ML models.

This study aims to explore the value of an ML model based on radiomics features and clinical features in predicting the clinical outcome of supratentorial sICH 90 days after surgery. We used a variety of algorithms to build models for comparison and selected the best algorithm for the task of prognosis prediction of SICH after surgery. We also explored whether the combination of clinical features and radiomics features could improve the prediction performance of the model. Shapley additive explanation (SHAP) plots were used to explain the contribution of model features in the prediction. We used multicenter data to validate the robustness of the model. To increase the methodological value of the manuscript, we followed the Transparent Reporting of a multivariable prediction model for Individual Prognosis Or Diagnosis (TRIPOD) statement, see Appendix A for details.

## 2. Materials and Methods

### 2.1. Study Patients

This is a multicenter retrospective study of 348 patients with spontaneous supratentorial intracerebral hemorrhage who underwent craniotomy evacuation of hematoma. There were 248 cases from Shanghai Tongji Hospital from March 2012 to October 2020, 50 cases from Shanghai East Hospital from October 2014 to December 2020, and 50 cases from Shanghai Xinhua Hospital from April 2016 to November 2020. All patients with sICH within this interval who met the inclusion criteria were collected.

Inclusion criteria: a. Preoperative imaging examination completed within 24 h after onset; b. Supratentorial parenchymal hemorrhage was confirmed by NCCT; c. Craniotomy evacuation of hematoma was performed.

Exclusion criteria: a. Traumatic cerebral hemorrhage, epidural hemorrhage, subdural hemorrhage, hemorrhagic transformation of ischemic cerebral infarction, cerebral hemorrhage caused by brain tumor, subarachnoid hemorrhage secondary to cerebral aneurysm or vascular malformation were excluded; b. Head CT did not meet the diagnostic criteria; c. Patients with incomplete clinical information.

The studies involving human participants were reviewed and approved by the Ethics Committee of Tongji Hospital (approval number: K-2020-021). Written informed consent for participation was not required for this study in accordance with national legislation and the institutional requirements.

### 2.2. Data Collection

Standardization of CT scanning parameters: routine axial head CT scan was performed from the skull base to the skull vertex with the orbitomeatal line (OML) as the baseline. The scanning conditions were set as follows: tube voltage 120 Kev, tube current 120 mA, slice thickness 5 mm, slice distance 10 mm, matrix 512 × 512.

We collected patients’ gender, age, and GCS on admission through the clinical medical record system. CT features included MLS, IVH, and deep ICH. The choice of these variables was decided based on the results of our previous study [7]. The term “Deep ICH” describes ICH that affects the thalamus, basal ganglia, internal capsule, and occasionally, the superficial cerebral lobe. At the cerebral falx, septum pellucida, and pineal gland, the MLS was measured. The supratentorial brain’s middle, upper, and lower layers were represented by them. The component with the greatest displacement distance was recorded when more than two parts were displaced simultaneously. The criterion for MLS is to measure the deviation of the cerebral falx center, pineal gland, and pellucid septum from the vertical axis connecting the anterior and posterior insertion points of the cerebral falx by more than 4 mm [13]. All images were independently interpreted in a blinded manner by two attending neuroradiologists, and in case of disagreement, a decision was made by one chief neuroradiologist.

### 2.3. Craniotomy Evacuation of Hematoma

All patients were treated according to the then-latest version of the Chinese guidelines for the diagnosis and treatment of cerebral hemorrhage, including the 2019 edition and the 2014 edition, and the 2007 Chinese guidelines for the prevention and treatment of cerebrovascular disease edited by Mingli Rao [4,14,15]. When a patient was in good health, did not have neurological symptoms that were progressively worsening, did not fit the criteria for surgical treatment, or was too elderly to tolerate surgery, they were given medicinal conservative treatment. When the hematoma volume was greater than 30 mL, the neurological symptoms were progressively aggravated or life-threatening brain herniation occurred. The chief or associate chief physician in charge of the patient should assess the suitability for surgery. The responsible chief physicians or deputy chief physicians handled all of the surgeries. A horseshoe incision was made on the part of the patient’s scalp that was closest to the hematoma after intravenous combination anesthetic was administered. Radially, the dural membrane was opened. In general, the cortex was cut along the axis of the brain gyrus until the hematoma area, and the hematoma was removed under a microscopy. The essential functional and vascular parts of the brain were avoided. An epidural catheter was inserted for drainage after the hematoma was removed, hemostasis was totally halted, and normal cranial closure was performed.

In patients with advanced cerebral herniation, those whose brain tissue collapse is not obvious after intraoperative removal of the hematoma or even above the bone window, and those whose brain tissue pulsation is not obvious or completely disappears after removal of the hematoma, decompressive craniotomy can be considered on the basis of hematoma removal. In this case, the dura is repaired with a piece of artificial dura, the cranial bone flap is removed, and a drainage tube is placed subcutaneously, drained through a skin poke hole, fixed to the scalp, and connected to a sterile drainage piece. The surgical instruments, gauze, and brain cotton are counted, the incision is disinfected, the temporalis muscle and scalp layers are sutured sequentially in layers, then disinfected, covered with a dressing, and wrapped. Typically, the drainage tube was taken out 3 to 7 days after the operation. One or both ventricles were drained simultaneously if the hematoma entered the ventricle.

### 2.4. End-Point

The purpose of this study was to predict mRS scores 90 days after surgery in patients with sICH. Dichotomous outcomes were defined as a score of 0–3 (good outcome) and 4–6 (poor outcome). In this study, a significant number of the patients had mRS scores between 4–6, and the patients who fulfilled the criteria for a craniotomy were typically in critical condition. Therefore, rather than 0–2/3–6, we classified the outcome of the mRS dichotomy as 0–3/4–6 [2,16,17,18]. The majority of the 90-day mRS data were gathered via telephone interviews, outpatient care, and clinical medical records. In phone interviews, patients were asked about their functional recovery 90 days following therapy (including whether they could walk unassisted and required assistance with daily activities). Scores ranged from 0 (no symptoms) to 6 (highest level of reliance or disability in daily activities) (death).

### 2.5. Patient Cohort

A total of 280 patients were finally enrolled due to the lack of follow-up information for some cases. The 215 cases of Shanghai Tongji Hospital were divided according to the ratio of 70% training set and 30% test set using a random sampling method. The training set n = 150; internal test set n = 65. In order to validate the performance of the model in different medical center datasets, we divided the external test set into two groups according to the data sources. Cases from Shanghai East Hospital were used as external test set 1 (n = 30), and cases from Shanghai Xinhua Hospital were used as external test set 2 (n = 35).

### 2.6. Image Segmentation and Preprocessing

The data are normalized according to the brain window (window width 80Hu, window level 40Hu). ICH lesions were manually segmented from NCCT images with the use of ITK-SNAP software (version 3.8.0, http://www.itksnap.org, (accessed on 20 February 2020)) [19], which was performed independently by two other attending neuroradiologists who were unaware of the patient’s clinical information. The segmentation results were reviewed and refined by two chief neuroradiologists. The CT image was resized; the size of the original image is 512 × 512 voxels, the size of the resized image is 256 × 256 voxels.

### 2.7. Radiomics Feature Extraction

Py-radiomics software package [20] was used to extract radiomics features in the region of interest (ROI) of the CT image corresponding to the mask, and a total of 108 radiomics features were extracted. There are 18 first-order features (mean, maximum, minimum, variance, percentile), 52 second-order features related to image texture, reflecting the extent and abruptness of grayscale intensity fluctuations in the image (Gray-Level Co-occurrence Matrix (GLCM), Gray Level Dependence Matrix (GLDM), Gray-Level Size Zone Matrix (GLSZM), Gray-Level Run-Length Matrix (GLRLM), Neighbouring Gray Tone Difference Matrix (NGTDM)), and 38 high-order features related to hematoma shape and three-dimensional size. The manually labeled image mask was used for radiomics feature extraction.

### 2.8. Feature Selection and Model Building

**Figure 1:**
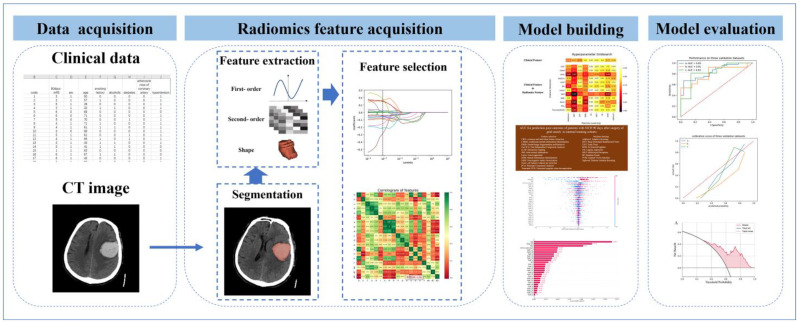
A flowchart of model development. Clinical and imaging data were collected, hematoma areas were delineated, radiomics features were extracted from areas of interest, and then feature screening and model construction were carried out to select the best model. Shap map was used to visualize feature contributions, and multicenter test set data were used to validate the model.

As shown in Figure 1, 12 feature selection algorithms were used to screen radiomics features. Nine ML postoperative prognosis prediction models using only clinical features and clinical features + radiomics features were constructed, respectively. The training set (n = 150) was used to train nine ML algorithm models, grid search was performed on different combinations of feature selection and ML algorithms for parameter tuning, and the model with the largest AUC was selected.

### 2.9. Model Test and Evaluation

The internal test set (n = 65), external test set 1 (n = 30) and external test set 2 (n = 35) were used to validate the ML prognosis prediction model with the largest AUC. The sensitivity, specificity, accuracy, and AUC of the model were calculated. A calibration curve was drawn to evaluate the consistency between the predicted results of the model and the actual observed values, and a Decision Curve Analysis (DCA) was drawn to evaluate the clinical practicability of the model. A Shap diagram was plotted for model interpretation. Figure 1 shows the radiomics workflow of this study.

### 2.10. Statistical Analyses

SPSS 20.0 statistical package was used to process the demographic data. Continuous data were represented as means [± standard deviation (SD)] or medians [± interquartile range (IQR)], whereas categorical variables were represented as numbers (percentage, range 0–100%). The Shapiro–Wilk test and histograms were used to evaluate the normality of the distribution. The Mann–Whitney U test was used to compare continuous variables between groups, while the Pearson Chi square test was used to analyze binary categorical data between groups. Missing data of continuous variables were filled with mean value. The difference was statistically significant at *p* ≤ 0.05. A heatmap was plotted to show the AUC results of 12 feature selection algorithms and nine machine learning models grid search. The ROC curve was drawn, and the sensitivity (SEN), specificity (SPE), accuracy (ACC), and AUC were calculated to evaluate the performance of the model. The task of training and test of the prediction model and statistical analysis were written in Python, version 3.6.

## 3. Results

### 3.1. Demography Data of Patients

We performed statistical analyses on the internal dataset and the external test dataset, respectively. As shown in Table 1, a total of 215 patients were included; 82 (38.1%) had a good outcome, and 133 (61.9%) had a poor outcome, among which the proportion of males (154, 71.6%) was significantly higher than that of females (61, 28.4%). The average age was (58.50 ± 13.82) years old, and the average age of the poor outcome group (62.51 ± 13.72) was significantly higher than that of the good outcome group (52.11 ± 11.39). ICH predominantly occurs in the basal ganglia and thalamus. In our cohort, 164 (76.3%) patients had deep ICH, and the proportion was higher in the poor outcome group (110, 82.7%). Seventy-five (34.9%) patients had MLS and 87 (40.5%) patients had IVH. MLS (60, 45.1%) and IVH (61, 45.9%) were more likely to occur in the poor outcome group. GCS on admission indicated that those in the poor outcome group (median 7; IQR, 5.00–9.50) were more likely to develop coma than those with good outcome (median 8; IQR, 6.75–13.00). Among the six clinical features, age (*p* < 0.001), deep ICH (*p* = 0.005), MLS (*p* < 0.001), IVH (*p* < 0.05), and GCS on admission (*p* < 0.001) were statistically different between the good outcome group and the poor outcome group. Table 2 presents the data distribution for the external test set with a total of 65 patients, of whom 30 had a good outcome and 35 had a poor outcome. The data distribution was generally consistent with the internal dataset. Age (*p* < 0.001), deep ICH (*p* = 0.005), and MLS (*p* < 0.001) were statistically different between subgroups.

### 3.2. Prediction Model Algorithm Selection

**Figure 2:**
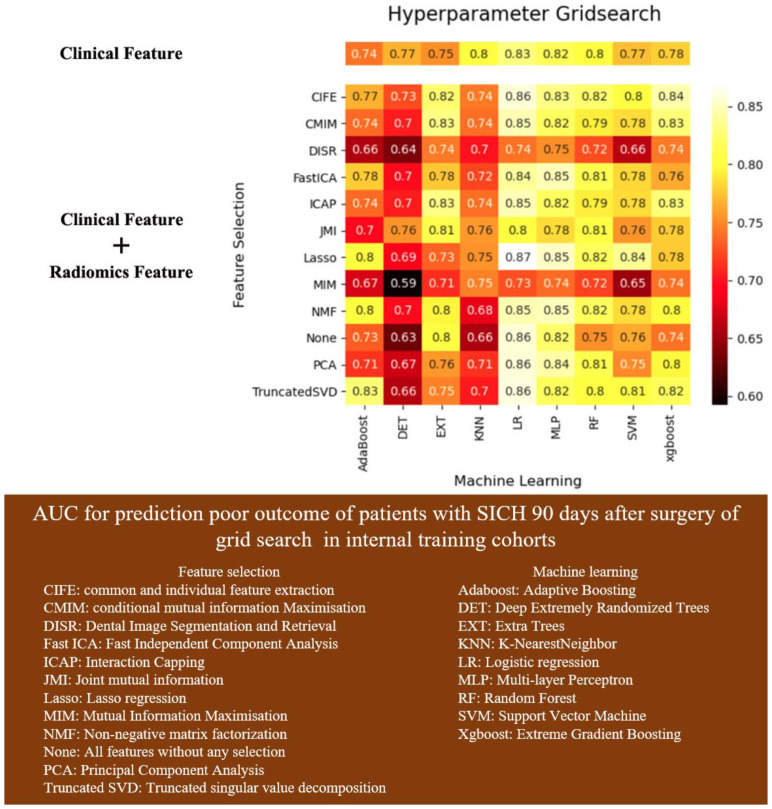
AUC for prediction poor outcome of patients with sICH 90 days after surgery of grid search in internal training cohorts.

The grid search results (Figure 2) of 12 feature selection algorithms and nine ML model based on clinical features + radiomics features showed that the combination of lasso regression feature selection and logistic regression model had the highest AUC: 0.87, better than nine prediction models using only clinical variables, AUC: 0.83.

### 3.3. Feature Selection and Model Interpretation

Lasso regression was based on the sparsity assumption, and features with 0 regression coefficients were eliminated, leaving 22 radiomics features and four clinical features. As shown in Table 3, among the 22 radiomics features, 16 second-order features represent the functional relationship between the gray values of the hematoma region (GLCM and GLDM represent the grayscale correlation of adjacent pixels, GLRLM provides information about the spatial distribution of consecutive pixels with the same gray level in one or more directions, in two or three dimensions, and GLSZM is based on a similar principle as GLRLM), but here the count of the number of interconnected groups of neighboring pixels or voxels (so-called regions) with the same gray level forms the basis of the matrix. The calculation can be performed for the distances of different pixels or voxels that defined the neighborhood. It can be calculated in two dimensions (eight neighboring pixels) or three dimensions (26 neighboring voxels); NGTDM quantifies the sum of the differences between the gray level of a pixel or voxel and the average gray level of its neighboring pixels or voxels within a predefined distance), four features are related to the shape and three-dimensional size of the hematoma (elongation, flatness, maximum diameter, area-volume ratio), and two first-order features (mean, minimum). First-order features are those calculated directly based on the pixel grayscale distribution of the original image, and their meaning can be understood based on the name.

**Figure 3:**
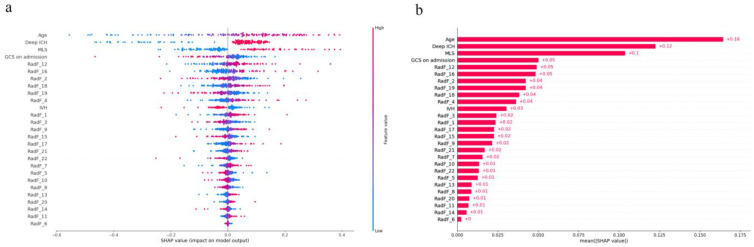
(**a**): Shap beeswarm plot of logistic regression prediction model. The Shap values of each feature were simply drawn by scatter. Different colors represent the relationship between the eigenvalues and the predicted influence, and the distribution of the eigenvalues is also shown. Red represents high eigenvalues, while blue represents low eigenvalues. (**b**): Shap global bar plot of logistic regression prediction model. The average value of Shap values of each feature is taken to make a bar chart, which shows the importance of each feature in the prediction. Note: For the convenience of presentation, RadF is the radiomics characteristic code, and its corresponding name is shown in Table 3.

The Shap values of each feature for each prediction were calculated. For each prediction, a positive Shap value indicates an increase in the risk of poor prognosis and vice versa. Figure 3a shows the distribution of Shap values generated by each feature when predicting all samples, and according to its color intensity, it can be seen that as the age increases, the probability of poor postoperative prognosis of patients with sICH increases. The smaller the GCS on admission, the more likely the patient is to have a poor prognosis. Patients with deep ICH and MLS are more likely to have a poor prognosis. In Figure 3b, the average of Shap values of each feature is made into a bar chart, which not only shows the contribution of the feature, but also shows its relative relationship. Other input model features included 22 radiomics features and IVH, but their contribution was lower than age, deep ICH, MLS, and GCS on admission. Higher-order features related to hematoma shape: RadF_2, RadF_4, and second-order features related to image texture: RadF_12, RadF_16, RadF_18, and RadF_19 had a greater weight in predicting the outcome and were higher than the clinical features: Intraventricular Hemorrhage. The second-order feature Gray Level Non-Uniformity Normalized was the most important radiomics feature.

### 3.4. Model Test and Evaluation

**Figure 4:**
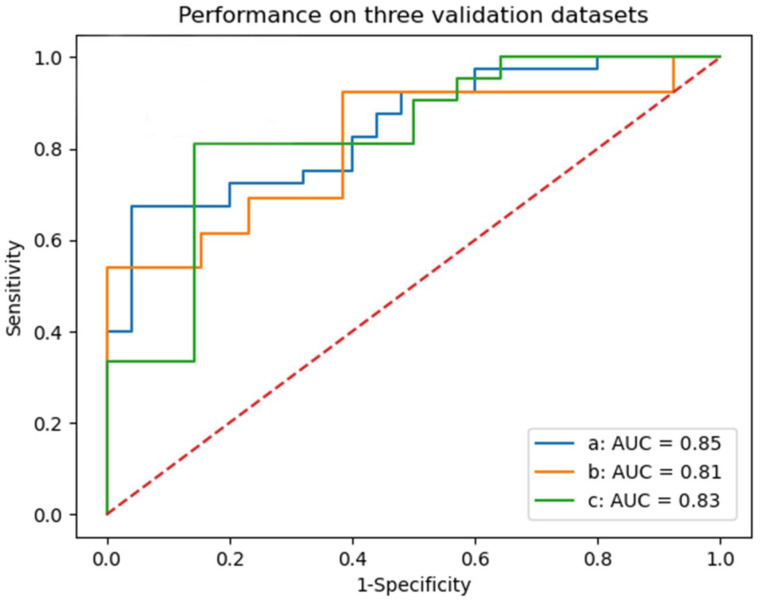
ROC curve of logistic regression model for predicting the prognosis of patients with sICH after surgery in three test sets. a: internal test set; b: external test set 1; c: external test set 2.

As shown in Figure 4, in the internal test set, the AUC of logistic regression model was 0.85 (95%CI, 0.75–0.94), and the AUC of external test set 1 and external test set 2 were 0.81 (95%CI, 0.64–0.99) and 0.83 (95%CI, 0.68–0.97), respectively. Table 4 shows the sensitivity, specificity, accuracy of the model, and the Youden index at the optimal cut-off.

**Figure 5:**
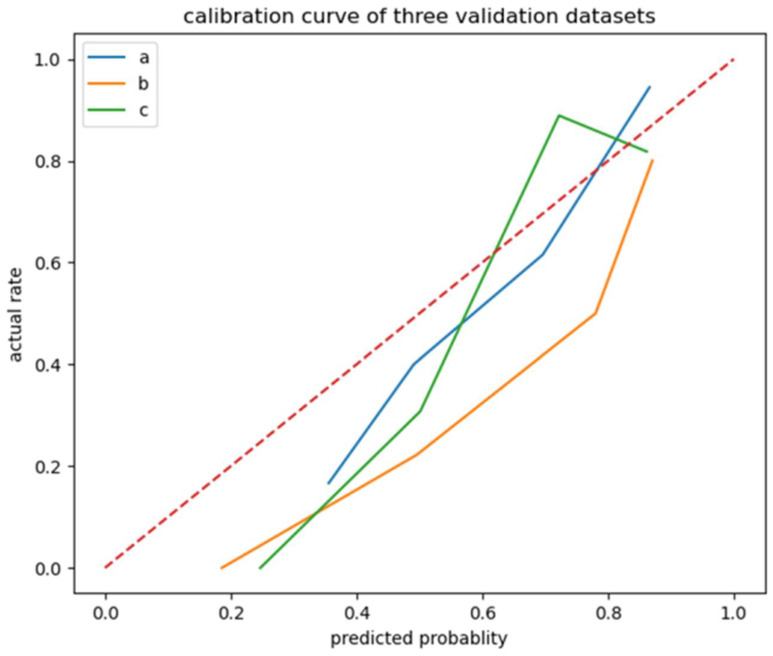
Calibration curve of logistic regression model. Line a: internal test set; Line b: external test set 1; Line c: external test set 2.

**Figure 6:**
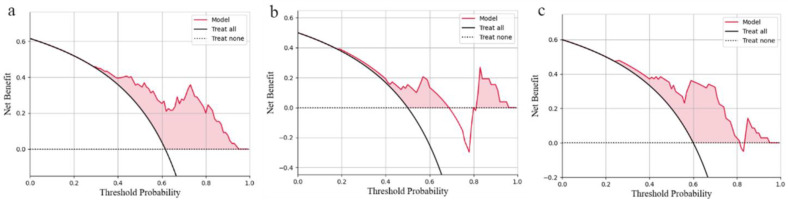
Decision Curve Analysis (DCA) of logistic regression model. (**a**): internal test set; (**b**): external test set 1; (**c**): external test set 2.

The calibration curve (Figure 5) of the logistic regression model revealed good predictive accuracy between the actual probability and predicted probability. The DCA curve (Figure 6) of the logistic regression model shows that in the internal test set, when the risk threshold is between 0.4–0.9, the net benefit of predicting poor outcome of patients with sICH after surgery is good. In external test set 1, when the risk threshold was 0.4–0.7 and 0.8–0.9, the net benefit of predicting poor outcome of patients with sICH after surgery was good, but the clinical net benefit was poor when the risk threshold was 0.7–0.8. In external test set 2, when the risk threshold was between 0.4 and 0.8, the net benefit of predicting poor outcome with sICH after surgery was good. The DCA curves indicate that the generalization ability of the model needs to be improved. On the one hand, in order to validate the performance of the model in different medical center datasets, we divided the external test set into two groups according to the data sources, which led to a lack of data volume; thus, we could not adequately evaluate the model performance, and this indirectly led to the lack of generalization ability of the model. On the other hand, data from different medical centers, even when the examination machines and examination parameters are the same, can lead to differences in data due to differences in the examination environment, which is a direct cause of insufficient generalization ability of the model, which is also the meaning of multicenter data testing in artificial intelligence research.

## 4. Discussion

### 4.1. Best Algorithm Suitable for the Task of Outcome Prediction of Patients with sICH after Surgery

Prognostic prediction for patients with sICH after surgery is helpful for clinicians to classify sICH patients with poor postoperative prognosis early, optimize treatment, achieve individualized precise treatment, and improve the overall prognosis of sICH patients. Previously, radiomics has been used to predict the prognosis of ICH, and considerable results have been achieved. Jawed Nawabi et al. [21] used the random forest algorithm to predict the clinical outcome mRS at discharge based on the radiomics features of ICH patients’ head CT, with an AUC of 0.80. The AUC of the model fused with ICH score was 0.84. Stefan Pszczolkowski et al. [22]. constructed a generalized linear model (GLM) based on radiomics features to predict adverse outcomes of ICH, and the best AUC was 0.78. The prediction performance of the model could be improved by combining radiomics features and clinical features.

Different from previous studies, our study focused on patients with sICH undergoing craniotomy evacuation of hematoma. By combining radiomics features and clinical features, we established a prognosis prediction model for patients with sICH 90 days after surgery. Twelve feature selection algorithms and nine ML models were used for grid search to select the model with the highest AUC, and were compared with nine ML models constructed using clinical features only. Finally, the best prognostic prediction model was selected: lasso regression feature selection + logistic regression prediction model with an AUC of 0.87 (95%CI, 0.80–0.92). Multicenter datasets were used to verify the best model, and both showed good prediction effect: AUC of internal test set: 0.85 (95%CI, 0.75–0.94), AUC of external test set 1:0.81 (95%CI, 0.64–0.99), AUC of external test set 2:0.83 (95%CI, 0.68–0.97). Compared with the single-center study, it was verified that our prediction model has good robustness. By comparing different features as model variables, we demonstrate that the combination of radiomics features and clinical features can improve the model performance. By comparing the models constructed by different algorithms, we found the best algorithm suitable for the task of prognosis prediction of patients with sICH after surgery, and avoided the limitation of using only one algorithm. This provides a very valuable reference for postoperative prognostic prediction studies of cerebral hemorrhage.

### 4.2. Logistic Regression Model and Lasso Regression

Logistic regression model is a classical dichotomous model. Based on linear regression, a sigmoid function is used to map the linear predicted value to the value interval of [0, 1], so as to complete the dichotomous prediction. It has the characteristics of simplicity, efficiency, strong integrity, and strong interpretability. In our study, the logistic regression model achieved the best results in the task of predicting the prognosis of patients with sICH 90 days after surgery.

Lasso regression is a model that adds L1 norm constraint term to the cost function of linear regression model. It screens variables and adjusts complexity through the control parameter lambda, which is widely used in the medical field. The model complexity is directly related to the number of variables in the model; the more variables, the higher the complexity of the model. More variables in the fitting can often give a seemingly better model, but also face the danger of overfitting. Based on the sparsity assumption, the lasso regression algorithm can screen out the variables that have important contributions to the model prediction, reduce the complexity of the model, improve the interpretability of the model, and prevent overfitting of the model.

The perceptron model is similar to the logistic regression model; both are binary linear models, the difference lies in their different activation functions. The perceptron uses the step function, while the logistic regression uses the sigmoid function; the step function is rougher for the classification results, non 0 that is 1, while the sigmoid function will output the results in the value range of 0–1, making the results have the ability of probabilistic interpretation.

### 4.3. Association between Radiomics Features and Hematoma

Among the 22 radiomics features selected by lasso regression, 16 second-order features, four hematoma shape-related features, and two first-order features were included. The Shap plot (Figure 3b) showed that second-order features had a higher contribution than shape features and first-order features in predicting prognosis. This indicates the importance of second-order features compared to first-order features and shapes-related features in the task of outcome prediction. In addition, higher-order features related to hematoma shape: RadF_2, RadF_4, and second-order features related to image texture: RadF_12, RadF_16, RadF_18, and RadF_19 had a greater weight in predicting the outcome and were higher than the clinical features: Intraventricular Hemorrhage. This suggests that radiomics features are more important than certain clinical features in the prediction of postoperative outcomes in patients with sICH. When the hematoma is in the bleeding active stage, the blood clots in the hematoma are high-density, while the continuous bleeding area shows mixed signs of relatively low density. When the patient has the risk of anticoagulation-related hematoma expansion, the hematoma can also show that there are relatively low-density areas in the high-density hematoma area [6,23]. Due to hematoma coagulation, serum absorption, and the evolution of intracellular hemoglobin, hematoma at different stages will show density differences on the NCCT, which are sometimes difficult to detect with the naked eye. Radiomics can accurately capture the gray difference of hematoma at different states, which can provide more imaging information for clinical analysis. This may also be useful for NCCT-based hematoma staging studies.

### 4.4. Association between Clinical Features and Poor Outcome

In the prediction model of this study, the top four features of contribution were age, deep ICH, MLS, and GCS on admission, which were all clinical characteristics and correlated with poor prognosis of sICH [24,25,26,27].

Age-related illnesses such as diabetes and cardiovascular disease, as well as a history of antithrombotic medication, are frequently present alongside aging and are all, in varied degrees, linked to a poor prognosis for ICH [28]. According to Rdholm et al.’s [24] study, getting older was significantly correlated with worse outcomes, such as death or severe impairment and ICH that was more severe. For the elderly patients with sICH, the operation should be fully prepared before operation as well as standardized nursing after operation. After surgery, older patients with sICH can benefit from holistic nursing mixed with humanistic nursing in terms of limb movement function, daily living activities, neurological impairment severity, and quality of life [29].

Previous research has shown that the involvement of deep functional areas of the brain is strongly associated with a poor outcome. This association may be due to the destruction of fiber bundles in these functional areas as a result of ICH, which would then result in the corresponding clinical symptoms. The in-hospital mortality of thalamic hemorrhage is higher than that of ICH of other parts of the supratentorial area [30]. Thalamic hemorrhage is more likely to spread to the brainstem and squeeze the ventricular, which could be fatal [31]. The odds of death, severe disability, and health index scale (EQ-5D) score were all increased by ICH affecting the thalamus and posterior limb of the internal capsule [26]. Research findings indicate that when minimally invasive surgery is combined with urokinase infusion therapy, the percentage of postoperative bleeding and case fatality can be reduced. Additionally, patients with basal ganglia hemorrhage can have better function in activities of daily living [32]. Therefore, the author believes that minimally invasive surgery should be carried out as far as possible according to hospital conditions when ICH involves functional nuclei in the thalamus, basal ganglia, internal capsule, and other deep brain regions.

MLS is caused by the mass effect of hematoma. Edema around hematoma will expand rapidly in the early stage of ICH, causing neuronal damage, and is closely related to poor prognosis [27]. Previous studies have shown that early clearance of the mass effect of hematoma can effectively reduce the occurrence and development of edema, reduce irreversible neuronal damage, and improve the prognosis of patients [33]. Therefore, the author believes that hematoma removal should be performed as soon as possible in patients with ICH when the MLS is greater than 4 mm to relieve the mass effect and the secondary brain injury caused by it.

In this study, the smaller the GCS on admission, the more likely the patients with ICH were to have a poor postoperative prognosis. GCS on admission represents the level of consciousness of patients on admission, which can reflect the severity of neurological impairment and is a reliable predictor of short-term mortality of ICH [34]. Our previous studies have found that surgery improved the clinical outcome of comatose patients [5]. According to previous studies, intracranial pressure monitoring should be performed for ICH patients with GCS score ≤ 8, and intracranial pressure should be maintained less than 20 mmHg. When the patient suffers from progressive deterioration of consciousness or coma, hematoma removal should be performed as soon as possible [35].

### 4.5. Limitations

There are still some limitations in this study. The volume of data is not large enough, which limits the model training and parameter tuning, and directly affects the model performance. In the tuning of hyperparameters, we used the method of grid search but did not tune all parameters of the model, only the key parameters, which may have some impact on the accuracy of the model, but this does not affect the conclusion. The limited data size of the test set also prevents the model from being fully evaluated. Our study is a retrospective study, and patient data were collected over a large time span, which is susceptible to selection bias. In the future, we will further expand the sample size, build a large-sample, multicenter, standardized ICH database, and carry out further research based on it.

## 5. Conclusions

The combination of clinical features and radiomics features using logistic regression models can improve the outcome prediction of patients with spontaneous supratentorial intracerebral hemorrhage 90 days after surgery. It is expected to achieve accurate preoperative assessment of postoperative prognosis of patients with sICH and improve treatment outcomes and clinical outcomes.

## Figures and Tables

**Table 1:** Baseline demographic, clinical, and radiological characteristics of training and test datasets.

Baseline Characteristics	ALL (n = 215)	Good Outcome (n = 82)	Poor Outcome (n = 133)	χ2/Z	*p* Value
Gender				3.81	0.051
Male (%)	154 (71.6)	65 (79.3)	89 (66.9)		
Female (%)	61 (28.4)	17 (20.7)	44 (33.1)		
Age mean (±SD)	58.54 (13.82)	52.11 (11.39)	62.51 (13.72)	2940.50	<0.001
Deep ICH (%)	164 (76.3)	54 (65.9)	110 (82.7)	7.96	0.005
MLS (%)	75 (34.9)	15 (18.3)	60 (45.1)	16.06	<0.001
IVH (%)	87 (40.5)	26 (31.7)	61 (45.9)	4.22	0.040
GCS score on admission medians (IQR)	8.0 (5.0–10.0)	8.0 (6.8–13.0)	7.0 (5.0–9.5)	3750.00	<0.001

**Table 2:** Baseline demographic, clinical, and radiological characteristics of external test datasets.

Baseline Characteristics	ALL (n = 65)	Good Outcome (n = 30)	Poor Outcome (n = 35)	χ2/Z	*p* Value
Gender				0.91	0.340
Male (%)	45 (69.2)	19 (63.3)	26 (74.3)		
Female (%)	20 (30.8)	11 (36.7)	9 (25.7)		
Age mean (±SD)	60.86 (11.05)	56.83 (12.73)	64.31 (8.05)	322.50	0.008
Deep ICH (%)	45 (69.2)	17 (56.7)	28 (80.0)	4.13	0.042
MLS (%)	24 (36.9)	7 (23.3)	17 (48.6)	4.42	0.036
IVH (%)	19 (29.2)	7 (23.3)	12 (34.3)	0.94	0.333
GCS score on admission medians (IQR)	9 (6.0–11.0)	9 (7.8–11.0)	9 (5.0–11.0)	418.50	0.154

**Table 3:** Radiomics characteristic codes and their corresponding names.

Code	Feature Name
RadF_1	original_ shape_ Elongation
RadF_2	original_ shape_ Flatness
RadF_3	original_ shape_ Maximum 2D Diameter Row
RadF_4	original_ shape_ Surface Volume Ratio
RadF_5	original_ first order_ Mean Absolute Deviation
RadF_6	original_ first order_ Minimum
RadF_7	original_ glcm_ Correlation
RadF_8	original_ glcm_ Idmn
RadF_9	original_ gldm_ Dependence Non-Uniformity
RadF_10	original_ gldm_ Large Dependence Low Gray Level Emphasis
RadF_11	original_ gldm_ Small Dependence High Gray Level Emphasis
RadF_12	original_ glrlm_ Gray Level Non-Uniformity Normalized
RadF_13	original_ glrlm_ High Gray Level Run Emphasis
RadF_14	original_ glrlm_ Short Run Low Gray Level Emphasis
RadF_15	original_ glszm_ Gray Level Non-Uniformity Normalized
RadF_16	original_ glszm_ Large Area Low Gray Level Emphasis
RadF_17	original_ glszm_ Size Zone Non-Uniformity
RadF_18	original_ glszm_ Size Zone Non-Uniformity Normalized
RadF_19	original_ glszm_ Small Area Low Gray Level Emphasis
RadF_20	original_ glszm_ Zone Percentage
RadF_21	original_ ngtdm_ Busyness
RadF_22	original_ ngtdm_ Coarseness

**Table 4:** Performance of logistic regression model in multicenter datasets.

Datasets	AUC	Sensitivity	Specificity	Yoden Index	Accuracy
Internal test set	0.85 (95%CI, 0.75–0.94)	0.68 (95%CI, 0.52–0.88)	0.96 (95%CI, 0.77–1.00)	0.64 (95%CI, 0.48–0.80)	0.77 (95%CI, 0.67–0.86)
External test set 1	0.81 (95%CI, 0.64–0.99)	0.92 (95%CI, 0.42–1.00)	0.62 (95%CI, 0.55–1.00)	0.54 (95%CI, 0.38–0.88)	0.73 (95%CI, 0.59–0.90)
External test set 2	0.83 (95%CI, 0.68–0.97)	0.81 (95%CI, 0.63–1.00)	0.86 (95%CI, 0.63–1.00)	0.67 (95%CI, 0.41–0.92)	0.80 (95%CI, 0.65–0.92)

## Data Availability

Not applicable.

## Appendix A

**Table A1:** TRIPOD Checklist: Prediction Model Development.

Section/Topic Item Checklist Item Page			
Title and Abstract			
Title	1	Identify the study as developing and/or validating a multivariable prediction model, the target population, and the outcome to be predicted.	1
Abstract	2	Provide a summary of objectives, study design, setting, participants, sample size, predictors, outcome, statistical analysis, results, and conclusions.	2
Introduction			
Background and objectives	3a	Explain the medical context (including whether diagnostic or prognostic) and rationale for developing or validating the multivariable prediction model, including references to existing models.	1–2
	3b	Specify the objectives, including whether the study describes the development or validation of the model or both.	1–2
Methods			
Source of data	4a	Describe the study design or source of data (e.g., randomized trial, cohort, or registry data), separately for the development and validation datasets, if applicable.	2–4
	4b	Specify the key study dates, including start of accrual; end of accrual; and, if applicable, end of follow-up.	2,4
Participants	5a	Specify key elements of the study setting (e.g., primary care, secondary care, general population) including number and location of centers.	2
	5b	Describe eligibility criteria for participants.	3
	5c	Give details of treatments received, if relevant.	3
Outcome	6a	Clearly define the outcome that is predicted by the prediction model, including how and when assessed.	4
	6b	Report any actions to blind assessment of the outcome to be predicted.	4
Predictors	7a	Clearly define all predictors used in developing or validating the multivariable prediction model, including how and when they were measured.	3–4
	7b	Report any actions to blind assessment of predictors for the outcome and other predictors.	3–4
Sample size	8	Explain how the study size was arrived at.	4
Missing data	9	Describe how missing data were handled (e.g., complete-case analysis, single imputation, multiple imputation) with details of any imputation method.	6
Statistical analysis methods	10a	Describe how predictors were handled in the analyses.	5–6
	10b	Specify type of model, all model-building procedures (including any predictor selection), and method for internal validation.	5
	10d	Specify all measures used to assess model performance and, if relevant, to compare multiple models.	5–6
Risk groups	11	Provide details on how risk groups were created, if done.	none
Results			
Participants	13a	Describe the flow of participants through the study, including the number of participants with and without the outcome and, if applicable, a summary of the follow-up time. A diagram may be helpful.	6
	13b	Describe the characteristics of the participants (basic demographics, clinical features, available predictors), including the number of participants with missing data for predictors and outcome.	6
Model development	14a	Specify the number of participants and outcome events in each analysis.	6
	14b	If done, report the unadjusted association between each candidate predictor and outcome.	none
Model specification	15a	Present the full prediction model to allow predictions for individuals (i.e., all regression coefficients, and model intercept or baseline survival at a given time point).	7–9
	15b	Explain how to the use the prediction model.	8
Model performance	16	Report performance measures (with CIs) for the prediction model.	10–11
Discussion			
Limitations	18	Discuss any limitations of the study (such as nonrepresentative sample, few events per predictor, missing data).	14
Interpretation	19b	Give an overall interpretation of the results, considering objectives, limitations, and results from similar studies, and other relevant evidence.	12
Implications	20	Discuss the potential clinical use of the model and implications for future research.	13–14
Other information			
Supplementary information	21	Provide information about the availability of supplementary resources, such as study protocol, Web calculator, and datasets.	none
Funding	22	Give the source of funding and the role of the funders for the present study.	14

We recommend using the TRIPOD Checklist in conjunction with the TRIPOD Explanation and Elaboration document.
